# Supplementary material for: Regulation of nitrogen availability results in changes in grain protein content and grain storage subproteomes in barley (Hordeum vulgare L.)
Source: PLoS One. 2019 Oct 16;14(10):e0223831. doi: 10.1371/journal.pone.0223831 (PMC6795425; doi:10.1371/journal.pone.0223831)
Supplement: Attachment — Submitted filename: Responses to Reviewers.docx [file pone.0223831.s002.docx]

**Responses to reviewer 1**

Comment 1. The English style should be revised by native English speaker.

Response 1: According to the reviewer’s suggestion, a native speak was invited to revise the manuscript.

Comment 2. P20 Line 382, the authors referred “A total of eight protein spots representing five hordein proteins were differentially expressed in Yangsimai 3 and Naso Nijo, which could contribute to the difference in GPC of mature barley seeds. Why? The authors should be further discuss.

Response 2: Thanks the reviewer for this content. The synthesis and accumulation of grain protein is highly dependent on the availability of nitrogen fertilizer, and it is difficult to understand the underlying control mechanisms of this. In the present study, we analyze the change of grain protein content and subproteome of seed protein in barley. The result showed that albumin and globulin are thus two subproteomes involved in GSP synthesis in grain seeds that are affected by nitrogen levels, a lot of differentially expressed proteins were observed. For example, GAPDH, Serpin-Z4, Z7 and so on. On the other hand, hordein fractions were affected N supply, we demonstrated the expression profiles of hordein and glutelin under low and high nitrogen conditions, and specific differentially expressed hordein proteins were identified. A total of eight protein spots representing five hordein proteins were differentially expressed in Yangsimai 3 and Naso Nijo, which could contribute to the difference in GPC of mature barley seeds at the protein level.

Comment 3. The introduction should explain the aim of the study.

Response 3: The purpose of this study as follows: In the present study, we report a comparative proteomics analysis of two cultivars (Yangsimai 3 and Naso Nijo) in high and low nitrogen conditions using two-dimensional gel electrophoresis (2-DE) and tandem Mass Spectrometry (MS). The main objectives were: (1) to investigate the difference in GPC and protein composition between low and high nitrogen-treated plants; (2) to obtain comparative information on the subproteomes of feed barley and malting barley under low and high nitrogen conditions; (3) to identify changes in protein composition and candidate proteins that increase GPC under high nitrogen conditions.

Comment 4. References should be in uniform format as required by Plos one publication.

Response 4: According to the reviewer’s suggestion, we have revised the manuscript according to the PLOS ONE's style requirements.

**Responses to reviewer 2**

Comment 1. The MS is a bit too descriptive and lacks major conclusion. The mechanisms underline the changes need to be discussed, i.e. many differentially expressed proteins were detected (the same as previously reported) but their roles in causing the difference between two varieties were not discussed.

Response 1: Thanks the reviewer for this comment. In the present study, the hordein fraction in barley grains was more sensitive to nitrogen and increased as the nitrogen level increased, which is the most stable factor for high GPC in feed barley. In addition, eight differentially expressed protein spots were detected in hordein proteome profile, which could contribute to the difference in GPC of mature barley seeds on the protein level.

Comment 2. Not sure if it's appropriate to compare the results with previously reported QTL.

Response 2: Thanks the reviewer for this comment. We have deleted QTL analysis in the revised manuscript.

Comment 3. Can the authors provide soil test results (how much N in the soil before N application)?

Response 3: According to the reviewer’s suggestion. We test the total N in the soil. Both barley cultivars were planted at the Yangzhou University Experimental Farm in autumn of 2014 in soil with a total N of 1.14g/Kg

Comment 4. Don't think they can use “biological" before replicates if their replicates are from a single experiment.

Response 4: Thanks the reviewer for this comment. A total of 100 seeds per genotype used for protein extraction and protein content measurement for each replication. Three biological replicates were performed in the present study.

Comment 5. A conclusion paragraph is needed at the end of Discussion.

Response 5: According to the reviewer’s suggestion. A conclusion paragraph was added in the revised manuscript.

Conclusion: In the present study, the GPC of mature grain was significantly higher in Yangsimai 3 than in Naso Nijo following nitrogen treatment. Hordein content was higher in both cultivars under high nitrogen conditions. Subproteome analysis revealed 152 differentially expressed protein spots on 2-DE gels. A total of 42 and 66 protein spots were successfully identified by tandem mass spectrometry in Yangsimai 3 and Naso Nijo under different nitrogen levels. Remarkably, the variation in B hordein content in Yangsimai 3 could have contributed to the higher GPC in Yangsimai 3 compared to Naso Nijo under different nitrogen availability conditions. Therefore, storage protein accumulation leads to GPC variation in feed and malting barley.
